# Provision of inpatient rehabilitation and challenges experienced with participation post discharge: quantitative and qualitative inquiry of African stroke patients

**DOI:** 10.1186/s12913-015-1057-z

**Published:** 2015-09-28

**Authors:** Anthea Rhoda, Natalie Cunningham, Simon Azaria, Gerard Urimubenshi

**Affiliations:** Faculty of Community and Health Sciences, University of the Western Cape Private Bag X17, 7535 Bellville, Western Cape South Africa; College of Medicine and Health Sciences, University of Rwanda, P.O Box 3286, Kigali, Rwanda

**Keywords:** Stroke, In-patient rehabilitation, Participation restrictions, Africa

## Abstract

**Background:**

The provision of rehabilitation differs between developed and developing countries, this could impact on the outcomes of post stroke rehabilitation. The aim of this paper is to present provision of in-patient stroke rehabilitation. In addition the challenges experienced by the individuals with participation post discharge are also presented.

**Methods:**

Qualitative and quantitative research methods were used to collect data. The quantitative data was collected using a retrospective survey of stroke patients admitted to hospitals over a three- to five-year period. Quantitative data was captured on a validated data capture sheet and analysed descriptively. The qualitative data was collected using interviews from a purposively and conveniently selected sample, audio-taped and analysed thematically. The qualitative data was presented within the participation model.

**Results:**

A total of 168 medical folders were reviewed for a South African sample, 139 for a Rwandan sample and 145 for a Tanzanian sample. The mean age ranged from 62.6 (13.78) years in the South African sample to 56.0 (17.4) in the Rwandan sample. While a total of 98 % of South African stroke patients received physiotherapy, only 39.4 % of Rwandan patients received physiotherapy. From the qualitative interviews, it became clear that the stroke patients had participation restrictions. When conceptualised within the Participation Model participation restrictions experienced by the stroke patients were a lack of accomplishment, inability to engage in previous roles and a perception of having health problems.

**Discussion:**

With the exception of Rwanda, stroke patients in the countries studied are admitted to settings early post stroke allowing for implementation of effective acute interventions. The participants were experiencing challenges which included a lack of transport and the physical geographic surroundings in the rural settings not being conducive to wheelchair use.

**Conclusion:**

Stroke patients admitted to hospitals in certain African countries could receive limited in-patient therapeutic interventions. With the exception of barriers in the physical environment, stroke patients in developing countries where resources are limited experience the same participation restrictions as their counterparts in developed countries where resources are more freely available. Rehabilitation interventions in these developing countries should therefore be community-based focussing on intervening in the physical environment.

## Background

Stroke is a major burden in developing African countries [1, 2]. An estimated 75 000 strokes occur in South Africa each year. Of these 25 000 stroke survivors die within the first month [3]. In Tanzania the overall crude yearly incidence of stroke was reported as 94 · 5 per 100 000 (95 % CI 76 · 0–115 · 0) and 107 · 9 per 100 000 (88 · 1–129 · 8) in the Hai and Dar-es-Salaam Regions respectively [4] Specific information about the burden of stroke in Rwanda is lacking. A recent study reported the rate of disability in adults as 10.4 % in the Bugesera Distrct and 19.6 % for the Muzanza District. Although the incidence and mortality of stroke have increased in these countries evidence-based management strategies have not been implemented [5]. This further increases the burden of the disease in these countries affecting the quality of life of the individuals who have experienced a stroke.

Rehabilitation post stroke has been found to be beneficial in facilitating recovery and improving quality of life [6, 7]. A multidisciplinary approach [8] which includes, physiotherapy, occupational therapy and speech therapy has been found to improve functional outcomes of individuals post stroke. Various lengths of hospital stay post stroke have been documented in the literature [9, 10] although the factor that has been found to improve outcomes relates not specifically to the length of stay but to the intensity of the rehabilitation received. When a greater intensity of treatment is provided early post stroke, it results in improved outcomes and recovery [8]. In developing African countries, the availability of health services is poor and inadequate [11]. The majority of stroke patient receiving out-patient rehabilitation at Community Health Centres in South Africa, only received between 1–5 Physiotherapy sessions which equated to a median number of 1.8 hours over a 6 month period [12]. Stroke patients who are admitted to in-patient rehabilitation facilities in South Africa [13] and Tanzania [14] also have limited follow-up treatment and contact with a Physiotherapist although they could benefit from these services [14]. According to the authors no published literature is available about the rehabilitation services provided to stroke patients in Rwanda. This limited availability and access to therapy services impacts on the quality of life and participation of people with disabilities which includes those who have experienced a stroke.

The challenges experienced by stroke patients after being discharged can be conceptualised within the World Health Organisation’s framework of functioning disability and health (ICF). The ICF is seen as a comprehensive framework and classification which provides a common language for all stakeholders including policymakers and persons who have become disabled [15]. The ICF is therefore useful for analysing the patient’s problems post stroke as well as assisting in enabling a systematic analysis of rehabilitation interventions [16].

Within the ICF framework, impairments and activity limitations are known to influence participation and quality of life which are seen as the ultimate goal of rehabilitation. The ICF could further be used to monitor rehabilitation outcomes from admission to reintegration into the community [17]. Included in this process is the identification of personal and social obstacles, as well as the management of interventions and measuring the effectiveness of rehabilitation interventions [17]. Although addressing participation restrictions is an important goal of rehabilitation, a number of barriers to participation have been identified. These include attitudes of others towards disabled people, lack of provision and access to services, problems with service delivery and inadequate financial resources [18].

The current paper focusses on the provision of inpatient rehabilitation and the post discharge challenges of stroke survivors in specific African countries.

## Methods

The study presents data collected in three African countries, namely South Africa, Tanzania and Rwanda. The settings for the different studies were all public hospitals which are funded by the state. The South African setting was the Eastern Cape with a population of 6 562 053 and reported the second lowest household income in the country. The study was conducted at the Provincial Hospital in the Eastern Cape with a bed capacity of 250 beds. The stroke patients are managed by a multidisciplinary team of professionals, ranging from the medical doctor, a neurologist (when available), nursing staff, and rehabilitation professionals which include the physiotherapist, occupational therapist and speech therapist. These rehabilitation professionals also provide outpatient sessions for those that require it.

The Tanzanian study was conducted in the Mbulu District in the Manyara Region, in the Northern Province of Tanzania. The setting for the Tanzania study was a referral hospital in the Northern Province of Tanzania. The hospital has a 450-bed and serves about 600,000 people who reside mainly in the Mbulu, Hanang and Babati Districts in the Manyara Region, and the Iramba district in the Singida regions which are mostly rural. Patients only receive institution-based physiotherapy services at the hospital as inpatients and at the time of data collection no other rehabilitation services were provided.

The setting for the Rwandese study was a district hospital, located in the Northern Province of Rwanda. It has a capacity of 409 beds. Stroke patients receive rehabilitation in the form of physiotherapy. There is neither outreach nor community-based rehabilitation services that are provided to stroke patients and these patients get institution-based rehabilitation services only as inpatients or outpatients. All interviewees, discharged from the district hospital lived in Musanze District, an area which is mainly rural, and where at least 91 % of the population is engaged in agriculture [19]. Musanze District has a total population over 380,000 having the highest density in the country: 770 persons per km2 [20]. Most families (about 65 %) live below poverty line [21]. Musanze is the most mountainous district in Rwanda [22], and there is therefore difficult geographical access and transport.

Qualitative and qualitative approaches were used to collect data [23]. The data relating to the provision of care was collected using a quantitative approach while the outcome data was collected using a qualitative approach. Information relating to the process of care was collected using a retrospective record review of a conveniently selected sample of patients admitted to the three hospitals. Records were reviewed where the diagnosis of stroke was made by a medical doctor based on the WHO definition of stroke [24]. Patients who had a hemiplegia as a result of a brain injury or tumour were excluded. To ensure sufficient data for statistical analysis, the South African charts were reviewed for a two year period, 1 January 2008 to 31 December 2009, the Rwandese charts for a four year period, 1 January 1st, 2005 and December 31st, 2008 and the Tanzanian charts for a 7 year period,1st January 2004 to 31st December 2010.

A validated document template was used to collect the quantitative data. Documented information that was captured included demographic characteristics (age, gender, marital status and employment status), medical characteristics (documented risk factors), stroke onset–admission interval, length of hospital stay, commencement of physiotherapy since admission, duration of physiotherapy and the total number of physiotherapy sessions.

Once ethical clearance and permission was obtained from the relevant parties, stroke patients who could be included in the studies were identified by reviewing hospital records. The data was collected by perusing the hospital and physiotherapy records of the patients. The data was collected by the main researchers, together with trained research assistants in their respective countries. Descriptive data was presented in the form of tables and figures displaying means, frequencies, percentages and standard deviations. A Pearson correlation between the number of physiotherapy sessions and the length of length of hospital stay was done. The significance level was set at *p* < 0.05.

### Challenges experienced by the participants post in-patient rehabilitation

The challenges experienced by the participants were determined qualitatively with a sample of those patients whose folders were reviewed as part of the quantitative part of the study. Although purposive sampling was planned for all the settings, the sample for the qualitative component for the South African setting was done conveniently because the initial purposively selected sample could not be recruited. The sample for the two other settings was recruited purposively. It was necessary to only consider subjects who were able to articulate their experiences and feelings, and therefore exclude individuals with communication or cognition problems. Participants, who needed assistance with at least one activity of daily living, as determined by the researcher, were interviewed.

Those participants, who were part of the quantitative retrospective study and were selected, were contacted telephonically to ascertain their willingness to be interviewed. The qualitative interviews were conducted at times and places convenient to the participants. Many participants preferred to have the interview conducted at their homes. The qualitative interviews were recorded by means of a digital voice recorder, but where the patients did not give their consent for the recording, detailed field notes were made during and immediately after the interview.

An interview guide was used to guide the interviews. The interview guide was developed based on the literature consulted [25–27]. The interview guide consisted of questions aiming to collect data which explored the challenges experienced by stroke patients while in the home or community setting. The interviews were conducted in a language in which the participant was fluent. The data was translated into English as part of the analysis process.

### Trustworthiness of qualitative data

Member-checking was the form of verification used. The stories shared by participants during the interviews were summarised and then retold by the researcher to the participants, in order to ensure that the researcher correctly understood the information provided during the interview. To enhance credibility of the qualitative data, the themes presented were illustrated with representative quotations from the transcribed texts [28]. To ensure that the qualitative data was confirmable, a peer examination was used by the researcher discussing the research process and findings with colleagues and experts with experience in qualitative research methods. For the same purpose, the study supervisor checked transcriptions, data reduction and analysis products (condensed notes), data reconstruction and synthesis products (thematic categories, interpretations) [29].

### Qualitative data analysis

The data was analysed thematically. The tape-recorded interviews were transcribed verbatim by the researchers. The transcriptions were read and compared to the audio tape recordings and field notes several times to verify accuracy [30]. Where necessary, a trained, multilingual translator translated the transcriptions into English and the researchers analysed those transcriptions to identify main patterns. Concepts were coded and then grouped into common themes. The analyses were conducted by the main researchers (NC, GU, SA) and peer reviewed to confirm themes by an independent person (AR).

Ethical clearance was obtained from the University of Western Cape, the Department of Health in the Eastern Cape, Tanzania Ministry of Health and Director of Haydom Lutheran Hospital respectively and the Rwanda National Ethics Committee. The participants were all given an information sheet which explained the aim of the study. Written informed consent was sought from all the participants. Participation was voluntary and participants were given the opportunity to withdraw from the study at any time. Participants were endured that information provided was handled confidentially and that pseudonyms will be used to protect participants' identities when results are published. The ethics number for the SA study was 10/1/23, the TZ study 10/9/25 and the Rwandan study was RW: No. 09/RNEC/2009.

## Results

### Demographic data

The demographic and process information is presented in Table 1. The sample for the retrospective survey consisted of 168 South African (SA) participants; 145 Tanzanian participants (TZ) and 139 Rwandese participants (RW). The South African sample consisted of slightly older participants. Although the length of hospital stay did not differ significantly between the different settings, the Rwandese participants were admitted at a longer post stroke period. Physiotherapy was received by almost all the participants in the South African setting, while less than 40 % of the Rwandese participants who were admitted to hospital received physiotherapy. A statistically significant relationship existed between length of hospital stay and number of physiotherapy sessions received for all three settings (*p* ≤ 0.0005).

**Table 1:** Demographic and rehabilitation process factors of the participants

	South Africa	Tanzania	Rwanda
Sample size number	168	145	139
Age: mean (SD)	62.6(13.78)	57.7(18.67)	56.3(17.4)
Ranges	20–90	19–80	17–92
Gender:			
Female %	59	52.3	53.2
Male %	41	47.7	46.8
LOS (days) (Standard Deviation)	7.38(5,10)	12.16 (4.1)	8.2 (10.18)
Range	2–41	0–180	0–172
TSO (days) (SD)	0.3 (1,04)	1.2 (0.42)	6.8 (18.348)
Range	0–7	0–3	0–180
% of participants receiving PT	98	67.5	39.6
Average number of physiotherapy sessions received	2	3	2
Ranges	1–22	1–32	1–24

*LOS* length of hospital stay, *TSO* time since stroke onset, *PT* physiotherapy

### Challenges experienced by the participants

Qualitative data was collected from 10 Rwandese (RP), 9 South African (SAP) and 10 Tanzanian (TP) participants.

Information relating to the challenges experienced by the participants with participation post discharge is presented within the Participation Model developed and verified by Barclay-Goddard [31]. This model identifies three broad constructs of participation which are related to accomplishment, role restriction and health efficacy.

### Accomplishment

Aspects that are highlighted in the model within this construct include social functioning, engaging in recreational activities, work, driving and usual activities.

The participants indicated that they experienced being *socially isolated*. The participants highlighted that they were restricted to their homes which could result in sadness and depression.

*“I cannot get where others are. I visit nobody. You understand it’s very hard … I just sit in the house till somebody comes to see me.” (RP).*

*“Family and friends do not come and visit me any more. I was last out of this house two years ago .... .I only sit at home .... I go nowhere. It makes me sad and depressed.” (SA).*

*I stay at home I cannot go anywhere …when I am in the wheelchair I cannot push myself because stones and stairs ……………………………. (TP).*

Participants also indicated that they could not participate in *leisure activities* as before. The leisure activities that were highlighted for these African stroke patients were mainly linked to engagement in religious or spiritual activities such as attending church. Participants became concerned about their role as Christians as a result of not being able to fulfil certain activities.

*“I go to church less than usual because I cannot reach there by myself … I have lost my Christianity Since I got sick, I went to church few times. It is only possible when my husband is there and brings me to church by car.” (RP).*

*“I am a catholic, but after getting sick I have never been to church for prayer. Because of my muscles weakness all the time I intended to try but I couldn’t manage to go. Sometime if my uncle is around, he used to help me by his car.” (TP).*

“*Since the stroke I do not go out much because I cannot walk far, less and less people ` come and visit me. I go to church now and then.” (SAP).*

The lack of ability to *engage in usual activities* was expressed by stroke patients in the different settings. The usual activities expressed below were mainly those related to domestic activities. Impairment of body functions was a factor that caused the inability to engage in usual activities and in previous roles.

*“I used to cook and clean my house now my situation of my hands is too heavy to hold, something and I always use two hands to hold bucket of water, look how I am holding, see my hand is too heavy … both my hands look weak and I can try but it takes me long time, my muscles is a bit stiff.” (TP).*

*“…Oh I cannot do anything with my hands…when other have gone to work…I try to see if I can clean the house but it very hard…I cannot do it (RW).*

*“I cannot do household tasks any more; I do not have the balance to stand on my own and work.”(SA).*

The *inability to drive* was not mentioned by many of the participants. A participant from Tanzania and one from South Africa, however, highlighted the challenges they experienced. They were dependent on others who were often not family members as well as on the public transport system which was often inconvenient. Once again, the impairments experienced by the participants influenced their ability to drive.

*“I have my own car, but my husband is not driving. We have to phone someone from KwaNobuhle to come and get us, if not possible, then we phone a cab. We plan our things according to his availability. It’s difficult with the cab – they don’t wait for you.” (SAP).*

*“I was driving, but you see now I couldn’t even touch a steering to drive … because of this spasm and my strength, even my legs look this contraction and I had lost my hope again on returning to drive.” (TP).*

The participants also expressed challenges regarding engaging in *remunerated occupation (work)*. They were concerned about not being able to support their families and felt uncertainty about losing their jobs. This was mainly expressed by the Tanzanian and the Rwandan participants. The South African participants were older and the majority was receiving an old age pension.

*“I used to teach and write, see how bad I am now…; my [ability to] return to the work is [difficult] in this situation.” (TP).*

*“I am not able to cultivate as I did before … you understand now I cannot get any money and I am being fed like a child…” (RP).*

### Restricted roles

The inability to resume roles such as family income-provider, protector, carer and parent, driver or decision-maker was also a difficult issue experienced by the participants. The participants discussed a change in role as a result of physical health problems. The inability to resume previous roles resulted in dependency and a lack of interest in previous activities.

*“As a man, I was the one to provide for my family and I had a young family at the time. I felt inadequate and in those days there were a lot of home invasions. I thought how will I protect them, it is my role to protect them … absolutely, I used to play football but … because of stroke I …have no interest on that at all.” (TP).*

*“Before I got sick I was a carpenter, but now I cannot do it to get money, and I have to get what I need from my children.” (RP).*

*“I have my own car, for 30 years I drove. Now, I can’t. It is difficult to sit in the passenger seat – it’s frustrating!” (SAP).*

### Health efficacy

Within this construct the aspects of perception of recovery and health are highlighted. The participants clearly indicated that they were worse since they had experienced a stroke and no perception of recovery was expressed. This lack of recovery resulted in participants expressing feelings of uselessness and sadness. Once again, the loss of movement was linked to a lack of recovery. Participants also linked the lack of recovery to being dependent on others for certain tasks.

*“Since the stroke I got worse, I feel useless, I cannot do anything for myself, I cry constantly.” (SAP).*

*“I hope that I’ll be able to walk but I doubt it very much, that I’ll ever get off on my own.”(TP).*

*“Since I became sick in 2004, I have never been able to walk and I always use a wheelchair to go somewhere.” (RP).*

Two constructs highlighted by the authors [32] as factors needing to be part of the participation model, were social support and the role of the physical environment. These aspects clearly arose from the qualitative interviews with the stroke patients included in this study.

### Social support

Some participants reported lack of support from their relatives since having a stroke, while others felt that the support was decreasing as time progressed. It became clear that participants were experiencing or perceiving that they were a bother to others, especially in situations where the assistance was being provided over long periods of time. The lack of social support also resulted in isolation of the stroke patients.

*“At the beginning, people were highly willing to help me. I was being helped by relatives and volunteers, but as it took a long time they became tired, and often they no longer come to visit me.” (RP).*

*“People/family does not want to help us by driving us around. I do not have good support from my family. When they see it’s me calling; they don’t answer the phone.” (SA).*

*“All my relatives have turned away from me … they do not care for me. I look after myself because I don’t have anyone to take care of me.” (TP).*

### Physical environment

The physical environment was highlighted as a factor which was a barrier to participation for these individuals. These barriers were detailed as stones, stairs and uneven grounds. A commonly mentioned occurrence was the participants’ ability to manipulate these features only with the help of another person.

They mentioned their physical as well as psychological inability to interact with these environmental features independently. Community features such as railings at stairs were described by participants as aiding their engagement in their environment. Environmental barriers related to the dependency of the participants on others in order to move around in the community.

*“I stay at home; I cannot go anywhere unless I have someone to assist me …Some ways are not good for me to walk, so sometimes I have to look for other ways which are practical for me. For example, uneven ways make my walking very difficult. So I must take a long way in spite of using a short one which is not practical to use for me.” (RP).*

*“I can climb the steps, but I need to have someone behind me; I am scared to climb the steps by myself; I am scared that I will get hurt“…I can only go into shops in town that have railings by the stairs, those I can climb, the others that don’t have, I will rather sit in the car.” (SAP).*

*“I couldn’t go up the road because I think it’s a bit steep. It would take ages getting up there and where do I go then? You’re at the main road, all the people rushing by. One knock and I’d lose my balance and I’m down again there, see. They say get out and walk but it’s easy for them to say get out and walk.” (TP).*

## Discussion

The aim of the paper was to present information relating to the process and outcomes of patients post stroke who were admitted to inpatient facilities in three East African countries.

### Provision of rehabilitation

The stroke patients admitted to inpatient facilities in this study had a lower mean age than what is reported for developed countries [26]. Stroke therefore remains a problem in younger individuals in African countries. Although this is the case, the cause of stroke in younger individuals has not been determined. The risk factor profile for these individuals is similar to what is recorded in older individuals in developed countries, with hypertension being the most prevalent risk factor. The lack of regular health checks and the lack of control of diseases such as hypertension could be the cause of stroke in younger individuals in these settings. There is therefore a need for health education and health promotion strategies to reduce the risk of stroke [32].

With the exception of Rwanda, stroke patients in the countries studied are admitted to settings early post stroke. This allows for implementation of effective acute interventions which could decrease stroke severity [33]. The late arrival of patients at hospital post stroke in Rwanda is, however, of concern. This is true both for the early intervention of medical management as well as for therapeutic interventions, both of which are recommended to commence early post stroke [33].

Stroke patients admitted to hospital in the Rwandese and Tanzanian settings were not guaranteed referral to physiotherapy. This is an indication of a lack of rehabilitation services which are strongly advocated in the management of stroke patients in order to improve outcome [8]. The short hospital stay and its link to the number of physiotherapy sessions in all the settings are alarming. It is clear that stroke patients are not admitted for rehabilitation but to ensure medical stability and are then discharged. What is of more concern is the fact that the patients are not followed up in the community. A number of challenges exists that could affect accessing outpatient settings that are attached to hospitals. These challenges include a lack of transport and of financial means to access transport [34]. The physical geographic surroundings in the rural settings are also not conducive to wheelchair use [35]. It therefore becomes clear that stroke patients in these countries receive minimal therapeutic interventions to assist with recovery due to a lack of access to rehabilitation services. A home or community-based rehabilitation approach would therefore be more appropriate for the management of these patients. The involvement and training of caregivers in the rehabilitation of the patient would be necessary and beneficial [36].

### Challenges experienced by the participants post discharge

When unpacked within the model of participation, it is clear that individuals from the three East African countries who had experienced a stroke, had problems in accomplishing the tasks they could perform previously. They also experienced a role change and perceived themselves to be ill, and as not having recovered from the stroke. They also experienced a lack of support from family and friends and physical environmental barriers. These findings were similar to findings of previous studies from both developed [37, 38] and developing countries [34, 35]. Individuals who had experienced a stroke in Canada [37] and an elderly population in Norway [38] experienced fewer barriers in the physical environment than were experienced by the African stroke sample.

In addition to the challenges experienced in the physical environment, the individuals stated that the lack of accomplishment of tasks and restricted roles they experienced was as a result of impairments or lack of the ability to conduct certain functional activities such as walking. Walking was found to be a factor which impacted on participation in a previous study [39]. Motor impairment, especially as it relates to the inability to use the upper and lower limbs, impacts on mobility and also on the ability to conduct tasks that need bilateral upper limb movements which are often needed in household tasks. In addition to the personal factors the external physical environment such as the uneven pathways and steep inclined roads found in rural areas also impacted on the individual’s ability to walk independently. This caused social isolation and a lack of participation of the individuals living in the rural parts of Africa. The physical environment also results in inaccessibility of rehabilitation services for persons who have experienced a stroke. Prior to their stroke individuals could walk the distances needed to access these services but post stroke they were unable to do and needed transport which was either too expensive or not available [35].

Lack of accomplishment and role restriction resulted in the individuals becoming dependent on others and feeling isolated. Participants clearly stated that they could not perform certain tasks unless being assisted by others. Not being able to engage socially also resulted in them being socially isolated. Furthermore, they become concerned when they could not fulfil their role as the breadwinner or caring for their families. Cultivating the land is a common practice in rural areas in developing countries. The participants from Rwanda and Tanzania that were employed were mainly cultivators. These individuals were not able to return to this type of job post stroke which not only affected them but their families as well as, expressed in the qualitative interviews. All these factors affecting individuals post stroke often result in psychological conditions such as mood changes and depression [40].

The aspect of social support is more often being considered as a factor which affects both participation and quality of life of individuals post stroke [40]. The participants of the studies conducted in Africa clearly indicated a lack of social support from families and friends. Lacking or fading social support could be a result of family having to care for others or support families in countries where poverty levels are high and people often have to cultivate land or carry out other tasks in order to support their families.

The authors would like to recommend the following in order to decrease the challenges to participation experienced by the individuals a Community-based rehabilitation (CBR) approach could be implemented in these African countries. CBR is seen as “A strategy for Rehabilitation, Equalization of Opportunities, Poverty Reduction and Social Inclusion of People with Disabilities [41]. Furthermore “CBR is implemented through the combined efforts of people with disabilities, their families and communities, and relevant government and non-government health, education, vocational, social and other services” [41]. Families and communities could therefore be mobilised to provide support to these individuals post stroke in order to assist with engaging in previous roles and therefore decreasing the participation restrictions experienced by these individuals. Efforts by both the communities and different sectors could also assisted in intervening in the physical surroundings of these individuals in an attempt to decrease the physical barriers to participation experienced.

### Limitations of the study

Data relating to in-patient rehabilitation was collected using a record review. This could have led to missing clinical data bias which could affect the results obtained. Information relating to the functional levels of the participants was not available. It is therefore not clear what the functional abilities were of the participants who provided the qualitative information.

## Conclusion

Stroke patients admitted to hospitals in certain African countries could receive limited in-patient therapeutic interventions with a short length of hospital stay. With the exception of barriers in the physical environment, stroke patients in developing countries where resources are limited experience the same participation restrictions as their counterparts in developed countries where resources are more freely available. The physical environmental challenges could lead to a lack of participation and access to rehabilitation services. Rehabilitation interventions in these developing countries should therefore be community-based including training and education of caregivers and intervening in the physical environment.
